# Elevated Admission Cardiac Troponin I Predicts Adverse Outcomes of Acute Type B Aortic Dissection after Endovascular Treatment

**DOI:** 10.3389/fsurg.2022.789954

**Published:** 2022-06-07

**Authors:** Kaiwen Zhao, Hongqiao Zhu, Lei Zhang, Junjun Liu, Yifei Pei, Jian Zhou, Zaiping Jing

**Affiliations:** ^1^ Department of Vascular Surgery, the First Affiliated Hospital of the Navy Medical University, Shanghai, China; ^2^ Department of Vascular Surgery, the First Affiliated Hospital of Qingdao University, Qingdao, China

**Keywords:** acute type B aortic dissection, cardiac troponin I, thoracic endovascular aortic repair, aortic-related adverse events, risk factors

## Abstract

**Background:**

There is a lack of evidence about the predictive role of serum cardiac troponin I (cTnI) on the long-term adverse outcomes of acute type B aortic dissection (aTBAD) patients after thoracic endovascular aortic repair (TEVAR). In this study, we identified whether cTnI was an independent risk factor of 5-year adverse outcomes for aTBAD patients after TEVAR.

**Methods:**

We reviewed consecutive aTBAD patients without previous heart disease who were admitted for TEVAR. The total study population was divided into the cTnI(+) group (≥0.03 ng/mL) and the cTnI(−) group (<0.03 ng/mL) according to the time-dependent receiver operating characteristic curve analysis. The differences in clinical characteristics, operative details and clinical outcomes were compared between the two groups.

**Results:**

There was no difference in age and male prevalence between the two groups. Compared with the cTnI(−) group, the incidence of chronic kidney disease was higher in patients with cTnI ≥0.03 ng/mL. In addition, the cTnI(+) group presented with more frequent premature beats and non-myocardial-infarction ST-T segment changes. In terms of laboratory examinations, white blood cell counts, neutrophil counts, serum D-dimer and serum fibrin degradation products showed an increase in the cTnI(+) group, while lymphocyte and platelet counts showed a decrease in these patients. Patients with elevated cTnI suffered from increased risks of 5-year aortic-related adverse events (hazard ratio, HR = 1.822, 95% confidence interval, CI: 1.094–3.035; *p* = 0.021) and all-cause mortality (HR = 4.009, 95% CI: 2.175–7.388; *p *< 0.001).

**Conclusion:**

Among aTBAD patients without previous heart disease, preoperative elevated cTnI identified patients at an increased risk of long-term adverse outcomes after TEVAR.

## The Number of IRB Approval

This retrospective study was approved by the Institutional Review Board of Shanghai Changhai Hospital (CHEC-Y2017, March 1, 2017). Written informed consent was obtained from all patients.

## Brief Summary

We conducted a retrospective study of 403 acute type B aortic dissection patients without previous heart disease after thoracic endovascular aortic repair to assess the relationship between cardiac troponin I and their outcomes. We identified that the elevation of cardiac troponin I was independently associated with a high risk of 5-year aortic-related adverse events and all-cause mortality.

## Introduction

Acute aortic dissection (aAD) is one of the most lethal conditions that is responsible for a number of aortic-related deaths (1). The incidence of aAD is about 3–4 per 100,000 individuals every year (2). According to the Stanford classification, aAD was categorized into acute ascending aortic dissection (aTAAD) and descending aortic dissection (aTBAD) (3). According to the literature, early thoracic endovascular aortic repair (TEVAR) could improve the rate of favorable aortic remodeling of aTBAD, which is protective against aneurysm formation (4). However, patients after TEVAR put up with the risk of unanticipated adverse events (5). Although multiple studies have identified the potential risks after TEVAR, it is a conundrum to find appropriate indicators to predict the prognosis of aTBAD patients after TEVAR.

Elevated admission cardiac troponin I (cTnI) has been proved to predict the short-term all-cause mortality of TAAD patients (6). However, there is little evidence about whether aTBAD could induce non-infarcted cardiac damage and whether aTBAD patients with higher preoperative cTnI suffer from worse outcomes after TEVAR.

In this context, we performed a single-center retrospective cohort study to investigate (1) the prevalence and clinical characteristics of aTBAD patients with elevated preoperative cTnI; (2) the difference in long-term outcomes between aTBAD patients with elevated preoperative cTnI.

## Methods

### Study Population

This is a single-center retrospective study of consecutive aTBAD patients receiving TEVAR in Changhai Hospital from the period January 1, 2003 to December 31, 2016. Ethics approval was obtained from the Institutional Review Board of Shanghai Changhai Hospital (CHEC-Y2017, March 1, 2017). Individual informed consent was obtained from each patient.

The exclusion criteria are as follows: obvious ST-segment elevations (≥0.2 mV) or depressions (≥0.2 mV) in two adjacent leads (21 cases); a history of recent myocardial infarction or undergoing percutaneous coronary intervention (12 cases); a history of atrial fibrillation or heart failure (HF) (47 cases); previous or concomitant procedures including thoracic aortic, abdominal aortic or cardiac surgery (5 cases); Marfan’s syndrome (3 cases); Takayasu arteritis (1 cases), subacute or chronic TBAD at the time of admission to our center (204 cases), end-stage renal diseases (6 cases) and patients without an examination of preoperative cTnI (18 cases).

### Study Protocol

The definition of aTBAD was according to the 2020 SVS/STS guidelines (7). The data of baseline characteristics, intra-operative details, medications at discharge, electrocardiograph, echocardiogram and computed tomographic angiogram (CTA) findings were retrospectively collected.

After admission, patients were administered with strict hypertension control according to the guidelines (4). Sedative and pain-relieving medications were given when necessary. An emergency TEVAR was conducted under the condition of malperfusion syndrome or hemodynamic instability (8). The timing of an elective procedure (the duration from onset to endovascular treatment: <24 h is the hyperacute phase, 1 day–14 days is the acute phase and 15–90 days is the subacute phase) was determined according to the experience of the surgeons (9). The hybrid approach was indicated for thoracic aortic diseases, including the aortic arch, and it had the potential to considerably improve results (10). The adjunctive technique with distal bare stent implantation had the advantages of reducing the partial thoracic false lumen thrombosis rate and the rates of new dissection and reintervention (11). The definition of a successful TEVAR was a complete exclusion of the proximal initial tear without any complications such as type I endoleak, type III endoleak and migration. Type II endoleaks were suggested to manage conservatively and monitor closely (1).

Serum cTnI was measured on admission and by Access Second-generation AccuTnI (Beckman Coulter, Brea, CA, USA). A cut-off value of cTnI was considered to be 0.03 ng/mL for the screening of acute myocardial infarction with 94% sensitivity and 87% specificity (12, 13). To examine the cut-off value of cTnI for predicting aortic-related adverse events (ARAEs), a time-dependent receiver operating characteristic (ROC) curve analysis was performed. The cut-off value of cTnI for 1-year all-cause mortality was 0.026, which was close to 0.03 (Figure 3A). Based on this result, patients were then assigned to the cTnI(−) group (<0.03 ng/mL) and the cTnI(+) group (≥0.03 ng/mL).

We collected the patients’ follow-up information through the electronic hospital and outpatient record system. Patients unable to visit their doctors were contacted by telephone. The follow-up deadline was February 2021.

Primary outcomes were defined as any ARAE at a 5-year follow-up: endoleak (type I and III), aortic dilation [unshrink type II endoleak and distal aortic segmental enlargement (11)], retrograde AAD (RTAD), rupture and malperfusion after TEVAR (11). Endoleak was described as the continuation of blood flow outside the lumen of the endoluminal graft but inside the aneurysm sac, most likely due to partial aneurysm exclusion from the circulation (14). Any new ascending, arching or descending dissection that was continuous with and proximal to the original presenting anatomy was considered a retrograde dissection (15). Rupture was defined as the extravasation of blood outside the confines of the aortic adventitia, which may be free or contained by the mediastinal pleura that surrounds the aorta (7). Malperfusion was defined as insufficient blood flow to the end organs as a result of dissection-related blockage (16). Secondary outcomes were MACCEs and all-cause mortality at a 5-year follow-up. Major adverse cardiovascular and cerebrovascular events (MACCEs) were defined as a composite of myocardial infarction, stroke and any coronary revascularization (17).

### Statistical Analysis

Data were presented as n (%) if the values were categorical variables, and mean ± standard deviations if the values were continuous variables. The Chi-square test or Fisher exact test was utilized to compare categorical variables, while student’s t-test was utilized to compare continuous variables.

Univariate associations between all clinical variables and ARAEs, MACCEs and all-cause mortality were calculated by Cox analyses. Variables with a *p* < 0.1 on univariate analyses were entered as candidate factors (Supplementary Material Table S1). Highly intercorrelated variables were excluded and multivariate Cox analyses were conducted utilizing the backward elimination method (*p* < 0.05). For each event of interest, the variance inflation factor ≥10 and clinical representability were taken into consideration. Decision curve analysis was used to evaluate the Cox regression models for ARAEs, MACCEs and all-cause mortality (18). Kaplan–Meier curves were used to investigate the differences of ARAEs, MACCEs and all-cause mortality between the two groups. An un-linear regression model was applied to examine the relationship between 5-year ARAEs and cTnI utilizing a smoothing function. All of the tests were 2-sided, and a value of *p* < 0.05 was accepted as statistically different. Statistical package R version 3.6.3 (R project, Vienna, Austria) was utilized to analyze the data.

## Results

### Baseline Characteristics

**Figure 1:**
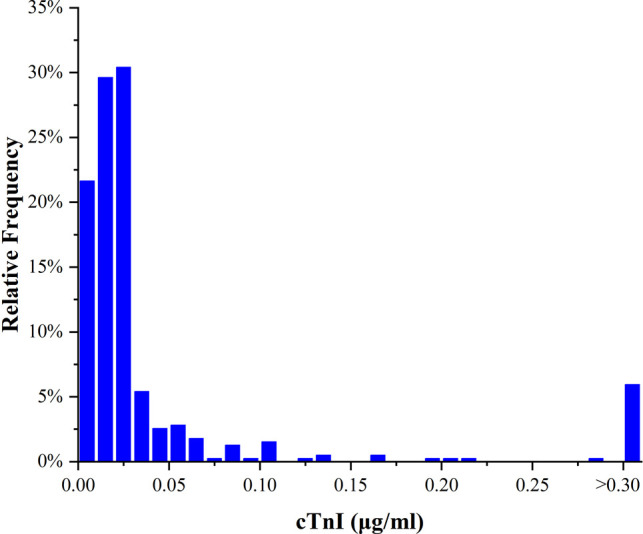
Frequency distribution histogram of cTnI in the study population (*n* = 403). cTnI, cardiac troponin I.

During the study period, 720 patients with TBAD received TEVAR in our center. After exclusion, 403 patients were eligible for study. The median values and ranges of cTnI in the cTnI(+) and cTnI(−) groups were 0.060 (0.030–7.300) *μ*g/mL and 0.012 (0.001–0.029) *μ*g/mL, respectively (*p* < 0.001) (Table 1). The frequency distribution of cTnI is presented in Figure 1. The mean follow-up time is 752 ± 622 days in the cTnI(+) group and 629 ± 618 days in the cTnI(−) group (*p* = 0.116). Table 1 demonstrates the baseline characteristics of the study population stratified with cTnI <0.03 ng/mL and ≥0.03 ng/mL. Among all patients, 317 (78.66%) had cTnI concentrations <0.03 ng/mL and 86 (21.34%) ≥0.03 ng/mL. Male prevalence was not significantly different between the two groups [cTnI(+) vs cTnI(−), 81.1% vs 82.6%, *p* = 0.754]. The mean age of the cTnI(+) and cTnI(−) groups was 59.8 ± 16.3 and 60.9 ± 12.4, respectively (*p* = 0.5). No differences were found in terms of the body mass index, smoking and drinking between the two groups (*p* = 0.783, 0.129 and 0.754 respectively). The differences in the incidences of hypertension, diabetes, previous stroke and chronic obstructive pulmonary disease (COPD) between the two groups were insignificant (all *p* > 0.05). The incidence of chronic kidney disease (CKD) in the study population was 5.7%, and the incidence of CKD was significantly higher in the cTnI(+) group than in the TnI(−) group [cTnI(+) vs cTnI(−), 11.6% vs 3.8%, *p* = 0.005]. There was also no difference in the presenting symptoms between the two groups (all *p* > 0.05). No difference was observed in the admission systolic blood pressure or diastolic blood pressure between the two groups (*p* = 0.475 and 0.541). Furthermore, 22 cTnI(+) and 48 cTnI(−) patients had myocardial ischemia-like ECGs (ST-T segment changes) (25.6% vs 15.1%, *p* = 0.023). The cTnI(+) group had more frequent premature beat manifestations (12.8% vs 4.7%, *p* = 0.007).

**Table 1:** Baseline characteristics of acute type B aortic dissection patients stratified with cTnI <0.03 ng/mL and ≥0.03 ng/mL

Variables	cTnI <0.03 ng/mL	cTnI ≥0.03 ng/mL	*P* value
	*N* = 317	*N* = 86	
cTnI, *μ*g/mL	0.012 (0.001–0.029)	0.060 (0.030–7.300)	<0.001
Age	60.9 ± 12.4	59.8 ± 16.3	0.5
Male	257 (81.1%)	71 (82.6%)	0.754
BMI	24.5 ± 3.5	24.3 ± 4.0	0.783
Smoking	173 (54.6%)	39 (45.4%)	0.129
Drinking	60 (18.9%)	15 (17.4%)	0.754
Comorbidities			
Hypertension	237 (74.8%)	69 (80.2%)	0.293
Diabetes	24 (7.6%)	5 (5.8%)	0.576
Previous stroke	19 (6.0%)	10 (11.6%)	0.073
COPD	41 (12.9%)	12 (14.0%)	0.804
CKD	12 (3.8%)	10 (11.6%)	0.005
Presenting symptoms			
Chest pain			
Anterior	189 (59.6%)	57 (66.3%)	0.261
Posterior	148 (46.7%)	41 (47.7%)	0.871
Shoulder pain	6 (1.9%)	1 (1.2%)	0.646
Abdominal pain	80 (25.2%)	19 (22.1%)	0.548
SBP on admission	136.8 ± 20.9	142.8 ± 29.5	0.475
DBP on admission	82.3 ± 11.3	84.5 ± 16.6	0.541
Pain severity			0.931
Mild	91 (28.7%)	26 (30.2%)	
Severe	90 (28.4%)	25 (29.1%)	
Worst ever	136 (42.9%)	35 (40.7%)	
ECG findings			
Premature beats	15 (4.7%)	11 (12.8%)	0.007
ST-T segment changes	48 (15.1%)	22 (25.6%)	0.023
LBBB/RBBB	36 (11.4%)	9 (10.5%)	0.816

*Continuous variables were presented as median (range) or mean ± standard deviation as appropriate. Categorical variables were presented as n (%). cTnI, cardiac troponin I; BMI, body mass index; COPD, chronic obstructive pulmonary disease; CKD, chronic kidney disease; SBP, systolic blood pressure; DBP, diastolic blood pressure; ECG, electrocardiography; LBBB/RBBB, left bundle branch block/right bundle branch block.*

### Detailed Information of CTA and Echocardiography

Table 2 presents the parameters of CTA and echocardiography in the two groups. The imaging results showed that arch type, diameters of maximum descending aorta, length of aortic dissection, proximal thrombosis of false lumen, malperfusion, diameters of ascending aorta, aortic root and left ventricular ejection fraction (LVEF) had no statistically differences (all *p* > 0.05). Meanwhile, pericardial effusion (12.8% vs 6.3%, *p* = 0.045) and pleural effusion (53.5% vs 33.1%, *p* < 0.001) were more common in cTnI(+) patients than those in cTnI(−) patients.

**Table 2:** Characteristics of CTA and echocardiography stratified with cTnI <0.03 ng/mL and ≥0.03 ng/mL

Variables	cTnI <0.03 ng/mL	cTnI ≥0.03 ng/mL	*p* value
	*N* = 317	*N* = 86	
CTA parameters			
Arch type			0.741
I	68 (21.5%)	19 (22.1%)	
II	79 (24.9%)	18 (20.9%)	
III	170 (53.6%)	49 (57.0%)	
Diameters of maximum descending aorta (mm)	41.5 ± 10.5	44.2 ± 12.6	0.544
Length of aortic dissection (mm)	414.9 ± 119.1	470.6 ± 109.9	0.116
Proximal thrombosis of false lumen			0.346
Patent	143 (45.1%)	46 (53.5%)	
Partial	103 (32.5%)	22 (25.6%)	
Complete	71 (22.4%)	18 (20.9%)	
Malperfusion			
Superior mesenteric arteries	1 (0.3%)	1 (1.2%)	0.321
Renal arteries	12 (3.8%)	7 (8.1%)	0.091
Common hepatic arteries	0	1 (1.2%)	0.055
Lower-extremity arteries	6 (1.9%)	1 (1.2%)	0.646
Pericardial effusion	20 (6.3%)	11 (12.8%)	0.045
Pleural effusion	105 (33.1%)	46 (53.5%)	<0.001
Echocardiography parameters			
Diameters of ascending aorta (cm)	3.4 ± 0.6	3.5 ± 0.5	0.619
Diameters of aortic root (cm)	2.3 ± 0.3	2.3 ± 0.4	0.336
LVEF (%)	62.1 ± 4.6	60.7 ± 7.4	0.252

*Continuous variables were presented as mean ± standard deviation and categorical variabl**e**s were presented as n (%). cTnI, cardiac troponin I; CTA, computed tomographic angiogram; LVEF, left ventricular ejection fraction; ULP, ulcer-like projection.*

### Endovascular Procedure and Antihypertensive Drugs

In the present study, all patients received TEVAR in the acute (14 days) or subacute (15–30 days) phase (7). During the endovascular procedure, 65 (16.1%) patients received the chimney technique, 46 (11.4%) the adjunctive procedure and 6 (1.5%) the hybrid approach (Table 3). Medications at discharge mainly included ACEI, ARB, β-blocker, CCB and diuretic. No obvious difference was found in the above antihypertensive drugs between the cTnI(+) and the cTnI(−) groups (all *p* > 0.05) (Table 3).

**Table 3:** Endovascular procedure and antihypertensive drugs stratified with cTnI <0.03 ng/mL and ≥0.03 ng/mL

Variables	cTnI <0.03 ng/mL	cTnI ≥0.03 ng/mL	*P* value
	*N* = 317	*N* = 86	
Procedure details			
General anesthesia	278 (87.70%)	69 (80.23%)	0.076
Timing of operation			0.008
Hyperacute phase	0 (0.0%)	3 (3.5%)	
Acute phase	231 (72.9%)	65 (75.6%)	
Subacute phase	86 (27.1%)	18 (20.9%)	
Chimney technique	48 (15.14%)	17 (19.77%)	0.301
Adjunctive procedure	39 (12.30%)	7 (8.14%)	0.282
Hybrid approach	3 (0.95%)	3 (3.49%)	0.114
Medications at discharge			
ACEI	20 (6.31%)	3 (3.49%)	0.317
ARB	104 (32.81%)	23 (26.74%)	0.283
β-blocker	139 (43.85%)	31 (36.05%)	0.194
CCB	169 (53.31%)	46 (53.49%)	0.977
Diuretic	45 (14.20%)	18 (20.93%)	0.127

*Categorical variables were presented as n (%). cTnI, cardiac troponin I; ACEI, angiotensin-converting enzyme inhibitor; ARB, angiotensin receptor blocker; CCB, calcium channel blocker.*

### Clinical Outcomes

**Figure 2:**
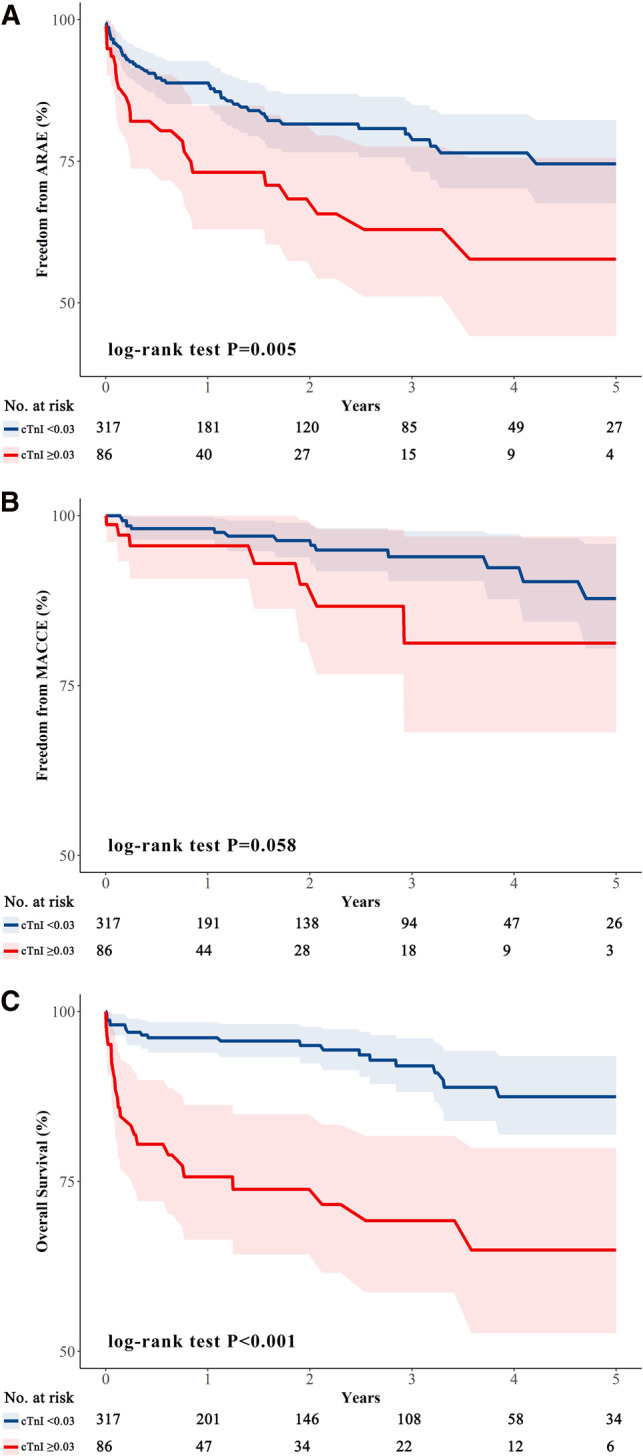
Kaplan–Meier curves. Survival curves for aTBAD patients after TEVAR, stratified with cTnI <0.03 ng/mL and ≥0.03 ng/mL. The differences in outcomes between the two groups were estimated by using the log-rank test. (**A**) The freedom from 5-year ARAEs between the two groups [cTnI(−) vs cTnI(+), 74.6% (67.6–82.3) vs 57.7% (44.1–75.6), *p* = 0.005]; (**B**) The freedom from 5-year MACCEs between the two groups [cTnI(−) vs cTnI(+), 87.8% (80.4–95.8) vs 81.2% (68.1–96.9), *p* = 0.058]; (**C**) The overall survival rates between the two groups [cTnI(−) vs cTnI(+), 87.5% (81.9–93.4) vs 64.9% (52.7–79.9), *p* < 0.001]. aTBAD, acute type B aortic dissection; TEVAR, thoracic endovascular aortic repair; cTnI, cardiac troponin I; ARAEs, aortic-related adverse events; MACCEs, major adverse cardiovascular and cerebrovascular events.

During the 1-year follow-up, the rates of missing data for overall survival, ARAEs and MACCEs were 32.1%, 35.2% and 41.4%. The 5-year missing data for overall survival, ARAEs and MACCEs were 85.4%, 80.4% and 93.1%. The Kaplan–Meier curves between the two groups are shown in Figure 2. Log-rank tests revealed a significant decreased rate of overall survival (*p *< 0.001) and freedom from ARAEs (*p* = 0.005) in the cTnI(+) group. Landmark analysis of MACCEs found that in the first three years, the difference between the two groups was significant [the cumulative incidence of 3-year MACCEs, cTnI(+) vs cTnI(−), 13.3% (2.0–23.4) vs 6.0% (2.3–9.6), *p* = 0.024].

**Figure 3:**
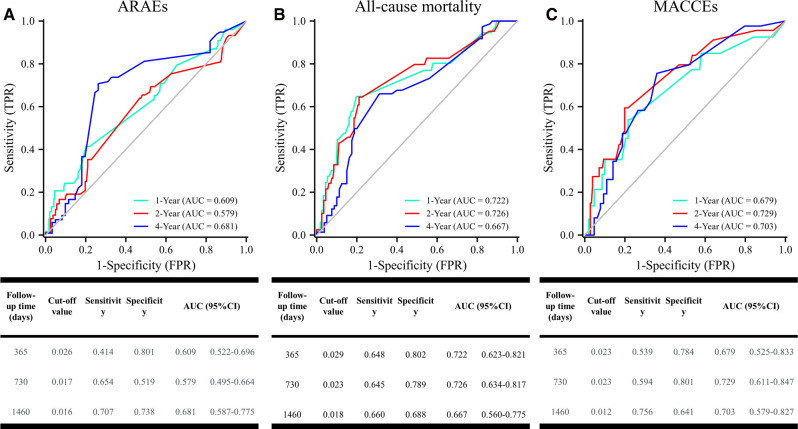
Time-dependent ROC curve analysis for cTnI predicting all-cause mortality. ROC, receiver operating characteristic.

Time-dependent ROC curve analyses were performed to investigate the predictive value of cTnI for ARAEs, all-cause mortality and MACCEs in aTBAD patients after TEVAR. The areas under the curve (AUC) of cTnI for predicting 1-year, 2-year and 4-year ARAEs were 0.609 (95%CI: 0.522–0.696), 0.579 (95%CI: 0.495–0.664) and 0.681 (95%CI: 0.587–0.775), respectively (Figure 3A). Figures 3B, C show the predictive ability of cTnI for all-cause mortality and MACCEs.

In the univariate Cox regression model, we analyzed the association between clinical variables and 5-year ARAEs, MACCEs and all-cause mortality (Table S1). After adjustment for cofounding factors, cTnI was confirmed to independently predict the 5-year ARAEs (hazard ratio, HR: 1.822, 95% confidence interval, CI: 1.094–3.035; *p* = 0.021). However, cTnI failed to predict the risk of 5-year MACCEs in the multivariable Cox regression model (HR: 2.579, 95%CI: 0.991–6.716; *p* = 0.052). We also found that cTnI (HR: 4.009, 95%CI: 2.175–7.388; *p* < 0.001) could independently predict the risk of 5-year mortality in patients with aTBAD after TEVAR.

**Figure 4:**
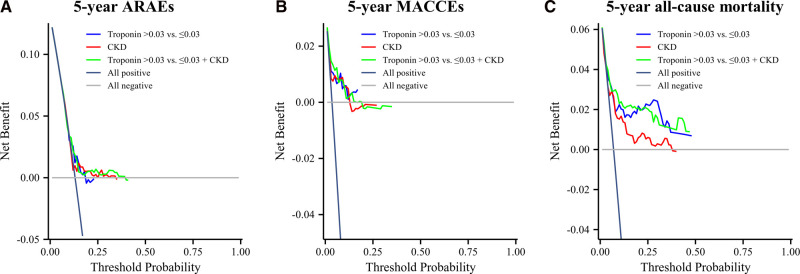
Decision Curve Analysis. (**A**) The decision curve analysis for 5-year ARAEs; (**B**) decision curve analysis for 5-year MACCEs; (**C**) decision curve analysis for 5-year all-cause mortality.

As shown in Figure 4A, the net benefit of the model including both cTnI and CKD was similar to that of the model of cTnI or CKD. A higher net benefit for 5-year all-cause mortality was achieved in the model with CKD than in the model with cTnI or CKD. (Figure 4B). There was no obvious difference in the net benefit among the three models (cTnI vs CKD vs cTnI + CKD) (Figure 4C).

**Figure 5:**
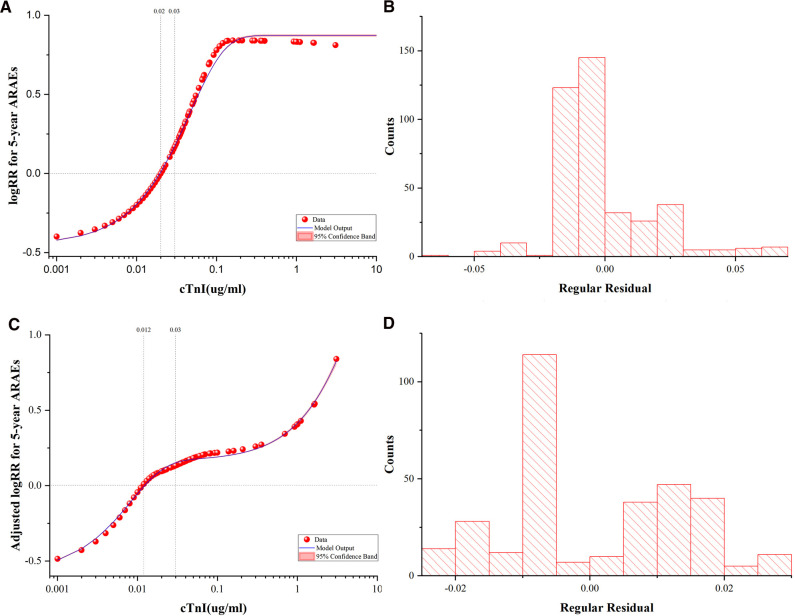
Fitted Curves and Residual Plots. The unadjusted and adjusted logRRs for 5-year ARAEs by different levels of cTnI. (**A**) Unadjusted logRR for 5-year ARAEs by the levels of cTnI. (**B**) Frequency histogram of residual plots in an unadjusted best-fit plot. (**C**) Adjusted logRR for 5-year ARAEs by the levels of cTnI. Adjusted curves were generated using the Cox model as the average of the model-based curves at observed profiles for the following covariates: age, sex, body mass index, chronic kidney disease, D-dimer and neutrophil counts. (**D**) Frequency histogram of residual plots in an adjusted best-fit plot. logRR, log relative risk; ARAEs, aortic-related adverse events; cTnI, cardiac troponin I.

The unadjusted and adjusted log relative risks (logRRs) for 5-year ARAEs by different levels of cTnI are demonstrated in Figure 5. This implied that the level of cTnI was positively correlated with a higher risk of ARAEs even after adjustment.

## Discussion

In the present study, we found that the elevation of cTnI concentrations on admission was associated with an increased risk of 5-year ARAEs and all-cause mortality in aTBAD patients after TEVAR (Table 4). However, there was no significant association between cTnI and MACCEs in a 5-year follow-up. There might be three reasons leading to this pattern. First, it may be attributed to the exclusion of patients with organic heart disease. Second, the landmark analysis indicated that in the first 3 years, the difference in MACCEs was significant between the two groups. The difference eliminated in the following 2 years may result from ageing in the cTnI(−) group. Third, MACCEs in the present study did not consist of HF (19). Interestingly, a community-based study showed that 1,089 asymptomatic elderly men with cTnI concentrations ≥0.03 ng/mL were associated with a hazard ratio of 5.25 for HF (20).

**Table 4:** Multivariable Cox analyses of the study population.

Variables	Hazard ratio (95% CI)	*p* value
5-year ARAEs (N = 82)		
cTnI		
<0.03	Reference	
≥0.03	1.822 (1.094, 3.035)	0.021
CKD	1.109 (1.025, 1.200)	0.011
5-year MACCEs (N = 21)		
cTnI		
<0.03	Reference	
≥0.03	2.579 (0.991, 6.716)	0.052
Diabetes	3.814 (1.214, 11.986)	0.022
Pericardial effusion	2.079 (0.681, 6.351)	0.199
5-year all-cause mortality (N = 43)		
cTnI		
<0.03	Reference	
≥0.03	4.009 (2.175, 7.388)	<0.001
Previous stroke	3.381 (1.603, 7.132)	0.001
CKD	1.208 (1.014, 3.114)	0.015
Diameters of ascending aorta	3.552 (1.582, 7.977)	0.002

*cTnI, cardiac troponin I; CI, confidence interval; CKD, chronic kidney disease; MACCEs, major adverse cardiac and cerebrovascular events; ARAEs, aortic-related adverse events*.

Our study used a time-dependent ROC curve analysis to determine the cut-off value of cTnI for ARAEs and all-cause mortality. Surprisingly, the cut-off values of cTnI for 1-year ARAEs and all-cause mortality were 0.026 and 0.029, which were very close to 0.03. In Li et al.’s study (21), the cut-off value of high-sensitivity cardiac troponin T for in-hospital death in aTAAD patients was 0.042, which was higher than 0.014. In our study, the major cause of death in TBAD patients the first year after TEVAR was aortic-related mortality (69.7%), which was consistent with that of Hysa et al.’s study (22). In this context, the cut-off value of cTnI 0.029 may predict 1-year aortic-related mortality after TEVAR.

cTnI is a biomarker of myocardial injury. It was confirmed that TAAD patients with elevated cTnI concentrations had a 4-fold higher risk of death compared with patients with lower cTnI concentrations (6). The proximal intimal flap of ascending aortic dissection may cover coronary ostia, which could reduce blood flow and induce acute myocardial infarction (23). What is worse, severe aortic regurgitation caused by TAAD may also result in ischemia of cardiomyocytes. Because the distance from the heart to the descending aorta is quite long, the damage to cardiomyocytes caused by aTBAD is often ignored. To our best knowledge, the present study is the first retrospective analysis to identify the potential predictive role of elevated cTnI in aTBAD patients after TEVAR, the possible mechanisms of which are as follows:

First, aTBAD patients presenting with higher cTnI concentrations may have cardiac-damage-related comorbidities such as heart diseases and CKD. For the purposes of cofounder control, we excluded those patients having any previous diagnosed organic heart diseases. The results in our study showed that the proportion of patients with CKD is significantly higher in the cTnI(+) group. A previous study has identified that cTnI may detect subclinical myocardial cell damage during hemodialysis (24). Left ventricular hypertrophy formation is also highly prevalent in hemodialysis patients with CKD, which may be responsible for elevated cTnI concentrations following the development of aTBAD (25). Moreover, high levels of homocysteine, lipid metabolic abnormalities and the accumulation of a large number of toxins (guanidine, phenols, indole, fatty amine) in patients with CKD trigger tremendous stimuli on myocardial cells (26), which may interfere with aortic remodeling and lead to the occurrence of ARAEs. Despite this, when adjusted for the presence of CKD in multivariate Cox analysis, cTnI remained an independent risk factor in 5-year ARAEs and all-cause mortality.

Second, recent research articles suggest that AD is a complex multifactorial disease, in which inflammation and thrombosis play important roles (27). Garcia et al. found that inflammation during sepsis may directly induce cardiomyocyte damage (28). When inflammatory factors are released, the expression of mitochondrial function–related genes and the pathway of cardiomyocyte apoptosis are activated, and cardiac cTnI will increase significantly (29). Specifically, interleukin-1 beta (IL-1β), IL-6 and IL-8 heighten inflammatory response, eventually resulting in exacerbated myocardial damage (30). Our results showed that the white blood counts, neutrophil counts and D-dimer were significantly higher in the cTnI(+) group than that in the cTnI(−) group. Collectively, inflammation may be a key factor underlying the association between myocardial damage and aTBAD.

Third, stress induces extensive biological changes, including the release of inflammatory cytokines and changes in relative organ functions. Accompanied by severe pain and hemodynamic instability, the onset of acute AD can activate the sympathoadrenal system and induce abundant catecholamine release (31, 32). A previous study illustrated that IL-6 is the dominant cytokine in response to acute stress (33). Psychological stress, featured with increased IL-6, is a confirmed risk factor in people with heart diseases (34–36). Therefore, stress may be another pathway for aTBAD inducing cardiomyocyte damage.

Hemodynamic instability caused by myocardial injury may result in adverse events in TAAD patients with elevated cTnI (6). Although we found that cTnI(+) patients were predisposed to have abnormal ECG presentation like premature beat and ST segment changes, there were no significant differences in terms of LVEF, systolic or diastolic blood pressure between the cTnI(+) and the cTnI(−) groups. This phenomenon indicates that a certain degree of cardiomyocyte damage may occur before any abnormal cardiac function and the appearance of hemodynamics. Subgroup analysis revealed that elevated cTnI was mainly associated with ARAEs consisting of malperfusion and rupture (Table 5). Local inflammation and thrombosis might play an important role in the mechanisms of malperfusion and rupture after TEVAR. Studies have shown that the recruitment and activation of neutrophils will lead to aortic rupture (37). Hyperinflammatory states will aggravate the degeneration of elastin, extracellular matrix and the apoptosis of smooth muscle cells, making the aortic wall more fragile and, thus, prone to avulse and rupture (38). Taken together, increased cTnI may have the ability to reflect severe hemodynamic conditions in aTBAD patients without previous heart diseases.

**Table 5:** Details of 5-year ARAEs stratified with cTnI <0.03 ng/mL and ≥0.03 ng/mL.

Variables	cTnI	cTnI	*p* value
	<0.03 ng/mL	≥0.03 ng/mL	
	*N* = 317	*N* = 86	
Cumulative incidence of ARAEs			0.005
1 year	11.2% (7.3–14.9)	26.9% (15.2–37.0)	
2 years	18.4% (13.1–23.4)	31.7% (18.6–42.6)	
5 years	25.4% (17.7–32.5)	42.3% (24.4–55.9)	
Cumulative incidence of type I/III endoleak			0.542
1 year	2.9% (0.9–4.9)	6.2% (0–12.7)	
2 years	6.3% (3.0–9.6)	9.0% (0.1–17.0)	
5 years	8.4% (4.0–12.5)	11.9% (1.3–21.3)	
Cumulative incidence of aortic dilation			0.519
1 year	3.1% (0.9–5.2)	2.9% (0–6.8)	
2 years	5.3% (2.3–8.2)	2.9% (0–6.8)	
5 years	8.2% (3.0–13.2)	2.9% (0–6.8)	
Cumulative incidence of RTAD			0.628
1 year	2.3% (0.5–4.1)	1.6% (0–4.6)	
2 years	2.8% (0.7–4.9)	1.6% (0–4.6)	
5 years	2.8% (0.7–4.9)	1.6% (0–4.6)	
Cumulative incidence of rupture			0.002
1 year	1.8% (0.2–3.3)	10.4% (2.6–17.5)	
2 years	1.8% (0.2–3.3)	10.4% (2.6–17.5)	
5 years	5.3% (0.9–9.5)	13.1% (3.7–21.6)	
Cumulative incidence of malperfusion			0.002
1 year	1.1% (0–2.3)	7.5% (1.0–13.7)	
2 years	1.6% (0–3.2)	7.5% (1.0–13.7)	
5 years	1.6% (0–3.2)	16.2% (1.8–28.5)	

*Differences in outcomes between the two groups were estimated by using the log-rank test. The brackets indicate 95% confidence interval. cTnI, cardiac troponin I; ARAEs, aortic-related adverse events; RTAD, retrograde type A aortic dissection.*

In summary, early preoperative monitoring of serum cTnI may facilitate risk stratification and follow-up of aTBAD patients after TEVAR. Cardio-preventive strategies are necessary to improve the survival rates of aTBAD patients after TEVAR.

## Limitations

In this study, there were some limitations to note. First, because the study was a single-center design and retrospective one, it was prone to selection bias. Second, the mean follow-up duration was slightly longer than 2 years; a longer follow-up period is needed for performing a more complete assessment.

## Conclusion

cTnI provided an important predictive value of the poor outcome in aTBAD patients after TEVAR, and cTnI ≥0.03 ng/mL was independently associated with increased 5-year ARAEs and all-cause mortality.

## Data Availability

The raw data supporting the conclusions of this article will be made available by the authors, without undue reservation.
