# Supplementary material for: Elevated Admission Cardiac Troponin I Predicts Adverse Outcomes of Acute Type B Aortic Dissection after Endovascular Treatment
Source: Front Surg. 2022 Jun 7;9:789954. doi: 10.3389/fsurg.2022.789954 (PMC9209658; doi:10.3389/fsurg.2022.789954)
Supplement: Supplementary file 1 [file Table_1_v1.docx]

**TABLE S1. Variables entered into multivariable Cox analyses**

| **Events of interest** | **Preoperative variables** |
| --- | --- |
| ARAEs | cTnI |
|  | Previous stroke |
|  | CKD |
|  | Pericardial effusion |
|  | Malperfusion of RA |
|  | Timing of operation |
|  | Diameters of ascending aorta |
|  | ACEI |
|  | Diuretic |
| MACCEs | cTnI |
|  | Diabetes |
|  | Previous stroke |
|  | LVEF |
|  | Pericardial effusion |
| All-cause mortality | cTnI |
|  | Previous stroke |
|  | CKD |
|  | Pericardial effusion |
|  | Timing of operation |
|  | Diameters of ascending aorta |

cTnI =cardiac troponin I; CKD =chronic kidney disease; RA =renal arteries; ACEI =angiotensin-converting enzyme inhibitor; LVEF =left ventricular ejection fraction; ARAEs =aortic-related adverse events; MACCEs =major adverse cardiac and cerebrovascular events.
